# Metagenomic Profiling of Soil Microbiomes and Resistomes in Arid Ecosystems of Kuwait

**DOI:** 10.3390/antibiotics15030294

**Published:** 2026-03-14

**Authors:** Ali A. Dashti, Leila Vali, Fiona Walsh

**Affiliations:** 1 Department of Medical Laboratory Sciences, Faculty of Allied Health Science, Health Sciences Center, Kuwait University, Safat 13110, Kuwait; 2 School of Education and Science, University of Gloucestershire, Cheltenham GL50 4AZ, UK; lvali@glos.ac.uk; 3 Department of Biology, Faculty of Science & Engineering, Maynooth University, W23 F2H6 Maynooth, Ireland; fiona.walsh@mu.ie

**Keywords:** ARGs (*tetA*, *aac(3)-Ib*, *sul1*, *qep*, *muxB*, *mexW*, *mexB*, *macB*), metagenomic analysis, novel detection of *ugd* in soil

## Abstract

**Background/Objective**: This study addresses a significant knowledge gap in the literature concerning antibiotic resistance genes (ARGs) in arid soils by employing metagenomic approaches to characterise their diversity, using Kuwait as a model environment. **Methods**: Soil samples were collected from two agriculturally managed sites (K1 and K3) and one coastal unmanaged site (K2), representing distinct ecological conditions. **Results**: Taxonomic profiling revealed notable variation in microbial communities at both the phylum and genus levels. Alpha diversity analyses based on the Chao1 and Shannon indices indicated that agricultural soils exhibited greater microbial richness and diversity than the coastal soil. Beta diversity analysis further demonstrated substantial differences in microbial community composition among the sites. Consistent with previous soil microbiome studies, ARGs such as *tetA*, *aac(3)-Ib*, *sul1*, *qep*, *muxB*, *mexW*, *mexB*, and *macB* were detected across the sites. However, the identification of distinct clinically relevant resistance genes, including *ugd*, *blaOXA-18*, *blaCMY-19*, *blaMOX-7*, *blaFOX-7*, *blaLRA-12*, and *novA*, suggests the influence of site-specific or extreme selective pressures. **Conclusions**: Several of the detected ARGs appear to be rare or previously unreported in soil environments. Although the sample size is too small to support broad generalisations, the detection of *ugd* in soil is particularly noteworthy, suggesting that soils may serve as reservoirs of polymyxin resistance, potentially undermining the effectiveness of polymyxin antibiotics.

## 1. Introduction

Characterising the diversity of the soil microbiome in terms of taxonomic and phylogenetic traits and relative abundances across different communities is important for understanding ecological systems [1]. Increasing evidence suggests that heat may influence the distribution and evolution of antibiotic resistance [2,3,4]. While prior studies have established a link between warm temperatures and both resistance in clinical pathogens and the presence of specific antibiotic resistance genes (ARGs) in natural ecosystems, metagenomic research on the environmental reservoirs of ARGs in the Middle East remains limited [5,6].

Kuwait’s soils are predominantly sandy and coarse-textured, which significantly limits their surface area and reduces both water and nutrient retention. Shallow calcareous hardpan layers, rich in calcium carbonates and quartz, often develop near the surface, impeding root penetration and restricting plant development [7]. These features, combined with persistent wind erosion, result in poorly developed soil profiles with minimal horizon differentiation and shallow rooting depths. The average water-holding capacity of Kuwaiti soils is approximately 7%, which allows only short periods of moisture availability. Conversely, infiltration rates are exceptionally high (50–100 cm/h), causing rainfall to bypass the root zone rapidly. Organic matter content is extremely low, reducing the soils’ nutrient retention and cycling capabilities. Key nutrients such as nitrogen, phosphorus, and potassium are present in suboptimal concentrations, necessitating regular supplementation for sustained plant growth [8].

From a microbiological standpoint, soil represents a critical environmental reservoir for ARGs, playing a pivotal role in the ecology of antimicrobial resistance (AMR). Investigating bacterial population dynamics across ecological scales is fundamental to understanding the evolution and dissemination of resistance [9]. Soil, particularly in agricultural environments, has been identified as a hotspot for the diversity and horizontal exchange of resistance genes [10]. Agricultural practices, including the use of antibiotics as growth promoters, veterinary treatments, and the disposal of pharmaceutical waste, contribute to the selection and spread of resistance determinants beyond their original ecological niches [11,12]. Molecular evidence confirms that certain ARGs are shared between environmental bacteria and human pathogens [13,14], and more alarmingly, the environment harbours novel ARGs with the potential to be acquired by clinical pathogens [15,16]. These findings underscore the importance of systematic environmental surveillance in tracking the emergence of ARGs in different geological terrains.

Despite the global urgency surrounding AMR, data from the Middle East, particularly concerning environmental reservoirs, remain scarce. Kuwait, characterised by an arid desert climate with seasonal extremes (summer temperatures > 50 °C and winter lows < 0 °C), limited freshwater availability [17], and frequent dust storms, provides a unique context for studying the environmental dimensions of AMR. Agriculture and livestock production are concentrated in isolated oases, creating localised hotspots for potential ARG accumulation. Dust storms further exacerbate microbial dispersal by transporting soil particles and associated microorganisms into urban and clinical environments. A notable historical example includes the Gulf War in 1991, during which multidrug-resistant *Acinetobacter* infections in injured soldiers were traced back to indigenous soil bacteria, highlighting the pathogenic potential of airborne microbes from arid soils [18].

However, the literature on ARGs in Middle Eastern soils remains extremely limited. A PubMed search conducted in December 2025 revealed only 40 publications indexed under (Middle East) AND (antibiotic resistance) AND (soil), and just five articles for (Kuwait) AND (antibiotic resistance) AND (soil) published between 2000 and 2025. Broader searches such as (bacteria) AND (Middle East) AND (soil) yielded 736 publications, while (bacteria) AND (Kuwait)) AND (soil) resulted in 121 articles from 1978 to 2025. Notably, searches combining (Middle East) AND (soil) AND (metagenomic) identified only 18 publications, and only 4 were retrieved under (Kuwait) AND (soil) AND (metagenomic) from 1990 to mid-2025.

This study aims to address this knowledge gap by focusing on metagenomic analyses of the presence and diversity of ARGs in arid soil ecosystems, using Kuwait as a case study environment. We acknowledge that these samples represent distinct environmental conditions and provide preliminary insights. Our objective is to improve understanding of how environmental factors, such as temperature extremes, affect ARG persistence and potential transfer to clinical settings. Understanding the environmental drivers of AMR, especially in underrepresented and climatically extreme regions like Kuwait, is critical for developing effective global strategies. The World Health Organisation’s One Health framework emphasises the interconnectedness of human, animal, and environmental health. This study integrates environmental microbiology and molecular biology to trace which ARGs circulate within soil environments. By focusing on Kuwait as a representative arid ecosystem, we seek to expand understanding of ARG dynamics and contribute valuable data to the global AMR surveillance landscape.

## 2. Results

### 2.1. Alpha and Beta Diversity

**Figure 1:**
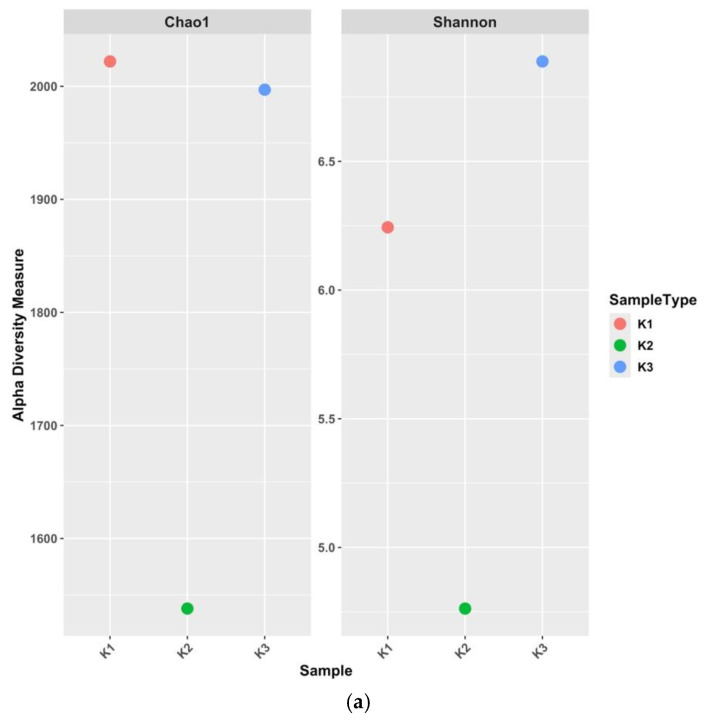

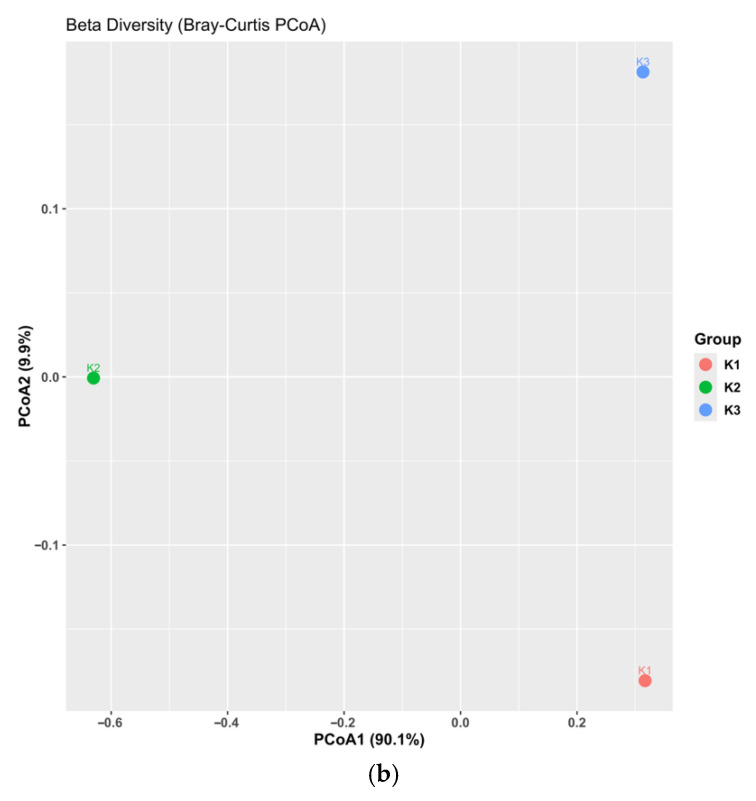
(**a**) Alpha diversity of K1, K2, and K3 soil samples. Alpha diversity metrics, Chao1 and Shannon indices, for three soil samples (K1, K2, and K3) (**b**) Beta diversity analysis using Principal Coordinates Analysis (PCoA). PCoA plot of K1, K2, and K3 soil samples.

**Figure 2:**
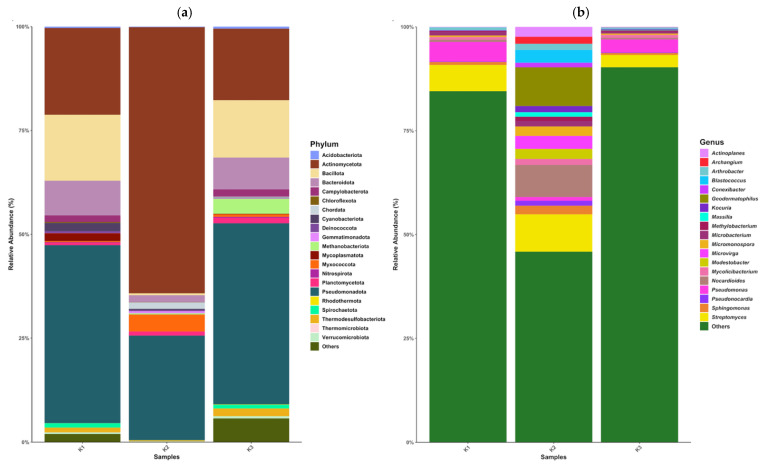
Taxonomic diversity in the soil samples (**a**) phylum level and (**b**) genus level.

The Chao1 index is shown in Figure 1, which estimates species richness. This indicates that K1 (low-intensity agricultural soil) and K3 (high-intensity agricultural soil) harbour higher genus richness compared to K2 (non-agricultural coastal soil), suggesting more diverse microbial communities in those samples. Similarly, the Shannon index (Figure 1), which accounts for both richness and evenness, shows higher values in K1 and K3, with K2 exhibiting the lowest diversity. Figure 2 shows that soil microbial communities differ substantially across the three sites, with K2 showing reduced taxonomic diversity.

**Figure 3:**
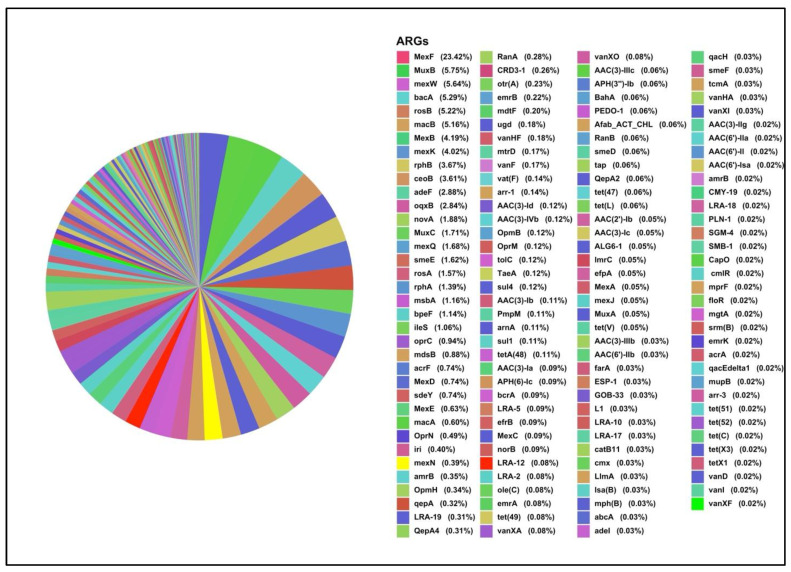
ARGs-wise resistance among microbes from the K1 soil sample.

Beta diversity analysis using Principal Coordinates Analysis (PCoA) based on the Bray–Curtis dissimilarity metric for the three soil samples (K1, K2, and K3) (Figure 3) illustrates the distinct microbial community compositions of the samples. The soil microbial communities are clearly separated into agricultural (K1, K3) and non-agricultural (K2), along with the first principal coordinate (PCoA1), which explains 90.1% of the variation. The second coordinate (PCoA2) explains an additional 9.9% of the variation, but the samples do not show substantial variation along this axis. This suggests that the microbial communities of these soil samples are highly distinct from one another, particularly K1 and K3 in comparison to K2, indicating significant beta diversity across the samples.

### 2.2. ARG-OAP Analysis for AMR

#### 2.2.1. AMR from the K1 Soil Sample

**Figure 4:**
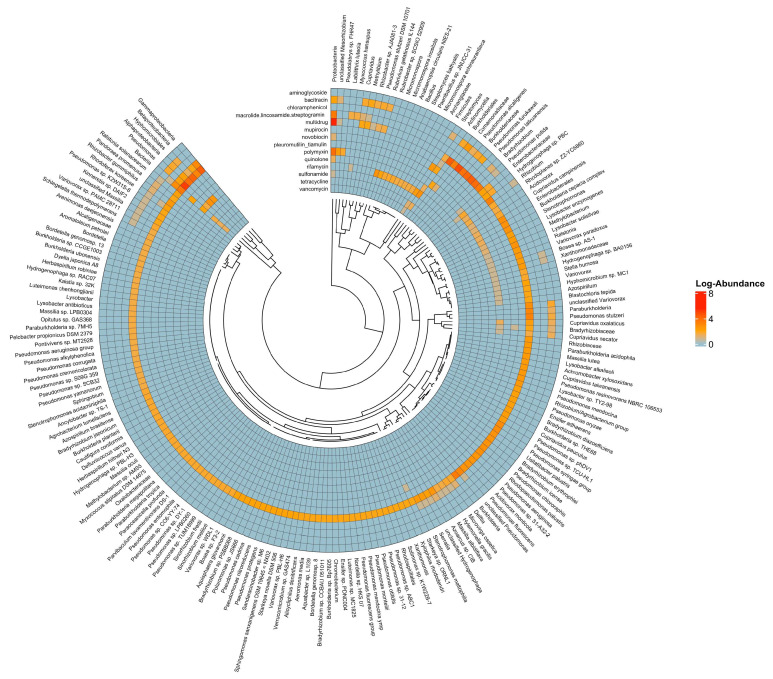
Class-wise resistance in the microbe-wise resistance from K1 soil sample. The colours scale, from red (high abundance) to blue (low abundance), represents log-transformed abundance. Dark blue (0 on a scale) means no gene detected.

The pie chart represents the relative abundance of various ARGs identified in the K1 sample (Figure 3), displaying a total of 143 unique ARGs. The largest proportion is dominated by *mexF* (23.42%), a gene typically associated with multidrug efflux pumps. Other highly abundant ARGs include *muxB*, *mexW*, *bacA*, *rosB*, *macB*, *mexB* and *qepA*, each contributing between 4% and 6% of the total ARG profile (Figure 4). The ARGs were predominated by efflux genes. Within this cohort of ARGs, *qepA*, is the most clinically relevant as it is a plasmid-mediated quinolone resistance gene. Also of clinical relevance is the detection of the β-lactamase genes: *bla*_CMY-19_ and *bla*_LRA_. An array of tetracycline resistance genes, e.g., *tet*A, and aminoglycoside resistance genes, e.g., *aac(3)-Ib*, as well as the sulphonamide resistance gene. Proteobacteria showed higher resistance, mainly mediated by multidrug resistance.

#### 2.2.2. AMR from the K2 Soil Sample

**Figure 5:**
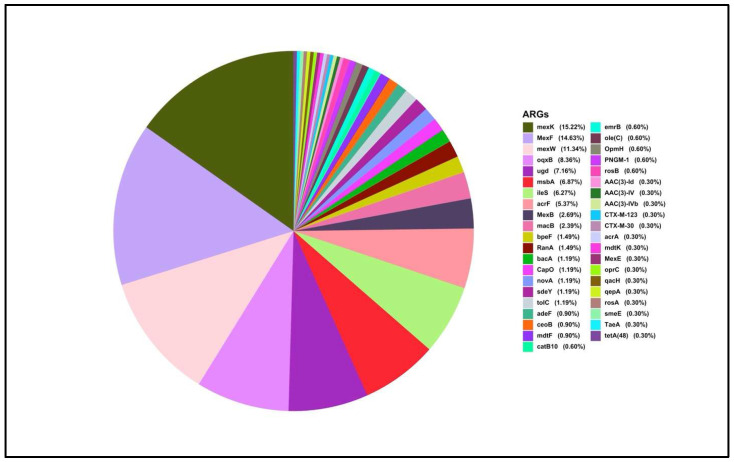
ARGs-wise resistance among microbes from the K2 soil sample.

**Figure 6:**
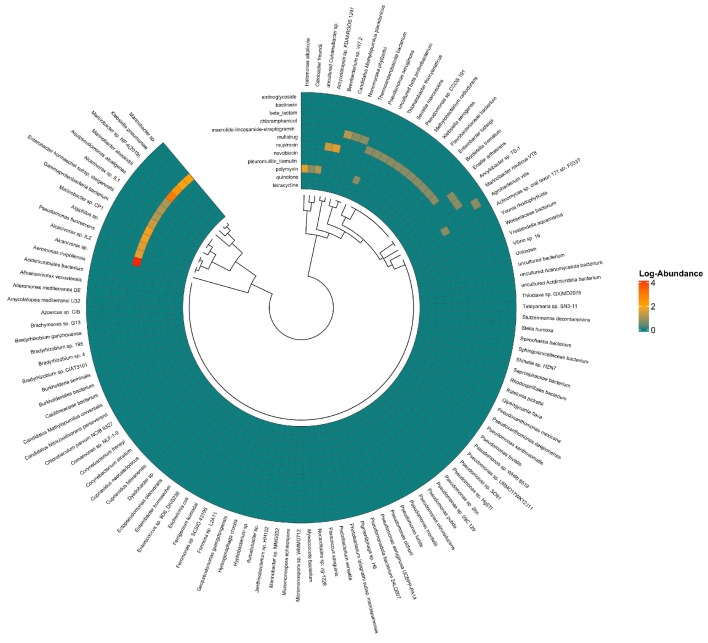
Class-wise resistance in the microbe-wise resistance from K2 soil sample. Colours scale from red (high abundance) to blue (low abundance) represent log-transformed abundance. Dark blue (0 on a scale) means no gene detected.

In the K2 soil sample (Figure 5 and Figure 6), most microbial communities showed resistance mainly through multidrug resistance mechanisms. The most found ARGs were *mex*K and *mex*F, both linked to multidrug efflux systems. Within the efflux gene group, the plasmid-mediated quinolone resistance gene, *qep*A, and resistance to third-generation cephalosporins via *bla*_CTX-M-30_ and *bla*_CTX-M-123_ were detected. Notably, the *ugd* gene was identified as the most abundant ARG related to polymyxin resistance. Of particular interest, compared with agricultural soil resistomes, is the markedly lower diversity of tetracycline resistance genes, with *tet*A being the only gene detected. No sulphonamide resistance genes were detected in K2. Among the microbial taxa, *Alcanivorax* demonstrated the highest level of resistance, predominantly mediated by multidrug resistance pathways.

#### 2.2.3. AMR from the K3 Sample

**Figure 7:**
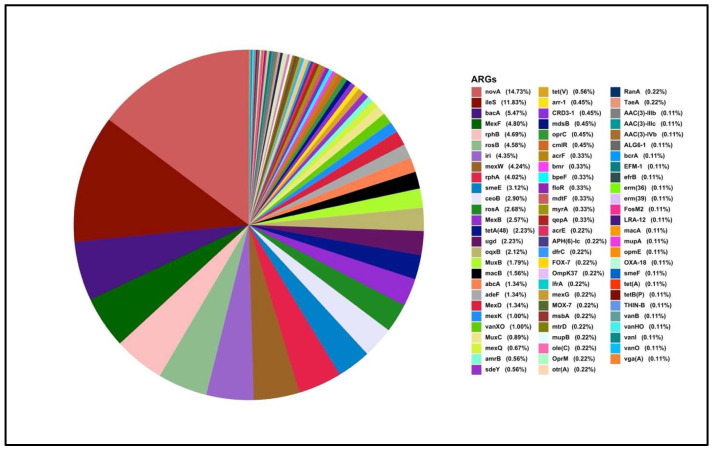
ARGs-wise resistance among microbes from the K3 soil sample.

**Figure 8:**
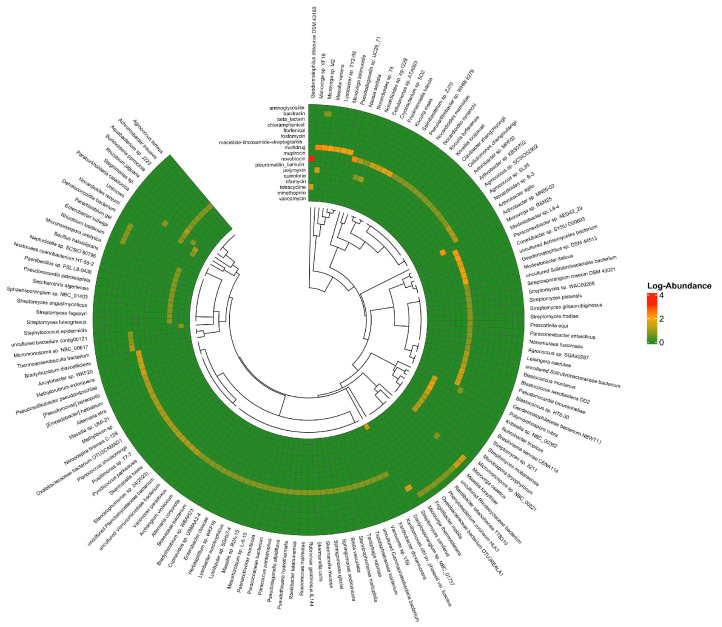
Class-wise resistance in the microbes from K3 soil sample. Colours scale from red (high abundance) to blue (low abundance) and represent log-transformed abundance. Dark blue (0 on a scale) means no gene detected.

From the K3 soil sample (Figure 7 and Figure 8), microbes exhibited resistance mainly through multidrug resistance, followed by novobiocin resistance. The most abundant ARG were *novA* and *ileS*, responsible for novobiocin and mupirocin resistance, respectively. The most observed multidrug resistance genes were *mexF* and *mexW*. As noted with the other two soil samples, the ARGs were predominated by efflux genes. Within this cohort of ARGs, the plasmid-mediated quinolone resistance gene, *qepA*, is most clinically relevant. Also of clinical relevance is the detection of the β-lactamases *bla*_FOX-7_, *bla*_OXA-18_, and *bla*_MOX-7_. Only *bla_LRA-_*_12_ beta-lactamase gene was detected. Similar to other soil microbiomes, an array of tetracycline resistance genes, e.g., *tetA*, and aminoglycoside resistance genes, e.g., *aac(3)-IIIb*, were also detected. Among the microbes, *Geodermatophilus* exhibited higher resistance to novobiocin.

Efflux pump genes (*mexF*, *mexW*) are present in all samples, indicating multidrug resistance mechanisms. K1: Dominated by *Pseudomonas*, a versatile genus with various resistance mechanisms. K2: Predominantly inhabited by *Alcanivorax*, a genus known for its hydrocarbon-degrading capabilities. K2 uniquely contains *ugd* (polymyxin resistance) which is rare in reported soils. K3: Shows a resistance profile with elevated levels of *novA* and *ileS*, correlating with the presence of *Geodermatophilus*, a genus known for high resistance to novobiocin.

Table 1 shows the key similarities and differences between the three soils. K2 uniquely contains *ugd* (polymyxin resistance), not detected in other soils, and *Alcanivorax* was the dominant taxa.

## 3. Materials and Methods

### 3.1. Sampling of Soil

**Figure 9:**
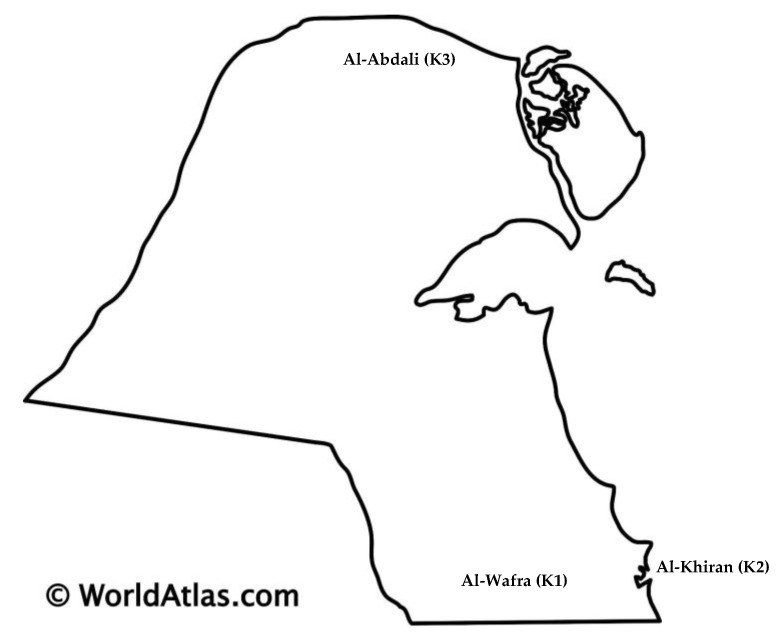
The map of Kuwait (https://www.worldatlas.com/maps/kuwait, accessed on 2 March 2026). The sampling sites of Al-Wafra K1, Al-Khiran K2 and Al-Abdali K3 are shown.

Soil samples (1 kg for each source) were taken (depth of 10 cm) into sterile glass flasks. From each sample, 1 to 5 g was taken for analysis, and the remainder was refrigerated and stored. Sampling was performed according to the methods described by Leski et al. (2011) [5]. The location of the sampling is shown in Figure 9. The sampling was performed at 45 °C in September 2024.

The K1 soil sample was collected from the Al-Wafra region, situated in southern Kuwait, where soil supports agricultural development and livestock production, albeit at a relatively lower intensity than the Al-Abdali area. The K2 soil sample was from Al-Khiran, a coastal area located in proximity to Al-Wafra in southern Kuwait and is not an agricultural site. The soil sample K3 was obtained from the Al-Abdali region, located in Northern Kuwait. This area is distinguished by intensive agricultural activity, a high density of farms, and a broad diversity of livestock, including ovine, bovine, caprine, and camelid species. The soil in Al-Abdali is generally classified as fertile and arable, supporting a wide range of agricultural practices. This region experiences relatively cooler temperatures compared to the southern areas of the country.

### 3.2. Metagenomics Sequencing

Metagenomic sequencing of the soil samples was performed on the Illumina platform (Novogene https://www.novogene.com). Gene prediction was performed on the assembled scaffolds using MetaGeneMark. The metagenomic reads were compared with the microNR database for taxonomy annotation. The functions of coding sequences were inferred by comparing them with databases. Antibiotic-resistance genes were annotated using the CARD database. Unigenes were compared against databases for insertion sequences, integrons, and plasmids. Quality assessment was conducted using FASTQC to evaluate sequence quality metrics such as per-base sequence quality, GC content, and adapter contamination. Following quality assessment, low-quality reads and adapter sequences were trimmed using the Trimmomatic pipeline with default parameters to ensure high-quality data for downstream analyses. Taxonomic profiling of the cleaned sequences was performed using the Kraken2 tool with the standard database [19], allowing for accurate classification of microbial communities present in the soil samples. For the identification and quantification of antibiotic resistance genes (ARGs), the ARG-OAP (Antibiotic Resistance Genes Online Analysis Pipeline) was employed, providing comprehensive insights into the resistome composition of the samples. The raw data can be accessed on request at report-https://doi.org/10.5281/zenodo.18980068 (https://connectglosac-my.sharepoint.com/:f:/g/personal/s2120274_glos_ac_uk/IgA2MrIIXw9aQpZdatz3dphqAYrcaK2mV1yeE8rlix5mkvU?e=y1mbAd, accessed on 2 March 2026) for K1, K2_EKDO250004138-1A_22ML2VLT4_L3_1.fa.gz (https://connectglosac-my.sharepoint.com/personal/s2120274_glos_ac_uk/_layouts/15/onedrive.aspx?id=%2Fpersonal%2Fs2120274%5Fglos%5Fac%5Fuk%2FDocuments%2FSequencing%2FK2%5FEKDO250004138%2D1A%5F22ML2VLT4%5FL3%5F1%2Efa%2Egz&parent=%2Fpersonal%2Fs2120274%5Fglos%5Fac%5Fuk%2FDocuments%2FSequencing&ga=1, accessed on 2 March 2026) and K3_EKDO250004140-1A_22ML2VLT4_L3_1.fa.gz (https://connectglosac-my.sharepoint.com/personal/s2120274_glos_ac_uk/_layouts/15/onedrive.aspx?id=%2Fpersonal%2Fs2120274%5Fglos%5Fac%5Fuk%2FDocuments%2FSequencing%2FK3%5FEKDO250004140%2D1A%5F22ML2VLT4%5FL3%5F1%2Efa%2Egz&parent=%2Fpersonal%2Fs2120274%5Fglos%5Fac%5Fuk%2FDocuments%2FSequencing&ga=1, accessed on 2 March 2026). In the event that the website is inaccessible or the link becomes invalid, the final report can be obtained from the corresponding author upon request.

### 3.3. Metagenomic Analysis

Raw metagenomic sequencing reads were assessed for quality using FastQC (v0.11.9). Adapter sequences and low-quality bases were trimmed using Trimmomatic (v0.39) with a sliding window approach (4 bp window, minimum average quality score Q20) and a minimum read length threshold of 50 bp. High-quality paired-end reads were de novo assembled into contigs using metaSPAdes (v3.13.0) with default parameters and multi-threading enabled. Taxonomic classification of quality-filtered reads was performed using Kaiju (v1.7.4) in greedy mode (-a greedy -e 5), which matches translated open reading frames against a comprehensive protein reference database. The classification utilised the “nr + euk” database (version 2023-05-10), which integrates protein sequences from bacteria, archaea, viruses, fungi, and other microbial eukaryotes, sourced from the NCBI non-redundant (nr) protein database [20].

### 3.4. ARG Annotation

ARGs were identified and quantified using the ARGs-OAP v2.3 pipeline (https://github.com/xinehc/args_oap, accessed on 2 March 2026), which aligns quality-filtered metagenomic reads against the Structured Antibiotic Resistance Gene (SARG) database (https://smile.hku.hk/ARGs/Indexing, version 2.0, accessed on 2 March 2026) [21].

The annotation process followed a two-step protocol. In the first step, a Perl script was used to detect sequences similar to ARGs and 16S rRNA genes, the latter serving as a normalisation reference. In the second step, ARG-like sequences identified earlier, along with associated metadata, were aligned against the SARG reference database using a separate Perl script executed on a local Linux platform. The alignment was performed with parameters set to a minimum of 25 amino acids in length, 80% identity, and an e-value threshold of 1 × 10^−5^. Contamination control is regulated within each of the pipelines and software tools referenced

### 3.5. Analysis and Visualisation

The resulting taxonomic Yang and ARG data were further analysed in RStudio (v4.2.3). Pie charts were generated using the ggplot2 v4.0.0 package [22], and heatmaps were created using the pheatmap package v 1.0.13 [23]. Additional R packages, including dplyr (v 1.1.0), reshape (v 1.4.5.), tidyr (v 1.3.2.), and readr (v 1.3.1.), were employed for data manipulation. The vegan package v 2.0-10 [24] was used for Bray–Curtis-based Principal Coordinates Analysis (PCoA).

## 4. Conclusions

Metagenomics offers a powerful tool to examine the composition and functional potential of soil microbiomes, providing insights into ARG diversity, abundance, and mobility. These data are essential for developing environmental protection policies and public health strategies aimed at mitigating the spread of resistance across ecosystems. This study examined the microbial taxonomic diversity and ARG profiles in three distinct soil samples (K1, K2, and K3). There are notable variations in taxonomic profiling, both at the phylum and genus levels, indicating the presence of diverse microbial communities. Alpha diversity analyses using the Chao1 and Shannon indices revealed that samples K1 and K3 exhibit higher microbial richness (higher Chao1) and diversity (higher Shannon) compared to sample K2. The lower Chao1 and Shannon indices in K2 suggest a reduced number and distribution of genera in that site’s ecosystem, reflecting stress and less favourable conditions [25,26].

Beta diversity analysis supported similar findings. Principal Coordinates Analysis (PCoA) based on Bray–Curtis dissimilarity showed clear separation among the three samples, particularly along the first principal coordinate (PCoA1), which accounted for 90.1% of the total variation. This strong separation suggests substantial differences in microbial community composition, with K2 differing markedly from K1 and K3. A large proportion of variation can be captured by PCoA1, showing the differences are derived from sample site variances [25]. The lack of significant variation along PCoA2 indicates that the primary driver of community differentiation is related to a single dominant gradient, which is in line with other studies showing that environmental site use and conditions are major drivers of soil microbial β-diversity [26]. The fact that PCoA2 explains only ~9.9% of variation also reflects that often the first axis captures most of the community-structure variation and subsequent axes’ smaller amounts [27]. Reduced K2 diversity might be due to the soil’s nutrient composition, organic matter content and salinity [28].

ARG-OAP analysis revealed the presence of numerous ARGs in all three samples, with multidrug resistance emerging as the predominant resistance mechanism, consistent with the global trend [29]. The K1 sample exhibited a wide array of ARGs (143 in total), with *mexF* representing the most abundant gene (23.42%). Other significant multidrug resistance genes included *muxB*, *mexW*, *mexB*, and *macB*. Efflux pump resistance systems are typically associated with *Proteobacteria* [30], the dominant phylum in this sample. Within this cohort of ARGs, the plasmid-mediated *qep*A, is the most clinically relevant gene conferring resistance to fluoroquinolone efflux pump *qep*. A gene has been previously detected in sewage-impacted wetland [31], farmland soil [32] and water [33]. Of additional clinical relevance is the detection of the AmpC β-lactamase gene *bla*_CMY-19_, which is believed to have evolved from *bla*_CMY-9_ under selective pressure from cephem antibiotics in clinical settings. This gene has been detected in *Klebsiella pneumoniae* and other members of the *Enterobacteriaceae* family [34]. Although CMY-type genes have been reported in environmental samples since 2010 [35], there are currently no specific reports documenting the presence of *bla*_CMY-19_ in soil.

We also identified *bla*_LRA_ (β-lactam resistance Alaskan) genes in K1 and K3 samples. *bla*_LRA_ were initially isolated from an unpolluted Alaskan sampling site soil [36], where external antibiotic exposure is scarce [37]. LRA group enzymes vary in class and active sites and represent all four Ambler classes. *bla_LRA-_*_12_ expresses a specific type of di-zinc-dependent metallo-β-lactamase (MBL), classified as Ambler class B, subclass B3 (chromosomally encoded enzymes from environmental bacteria), that confers significant resistance to a broad range of β-lactam antibiotics, including carbapenems, penicillins, and cephalosporins [38], highlighting that ARGs exist in the natural environment independent of human antibiotic use. When *bla_LRA-_*_12_ gene was expressed in *E. coli* in a laboratory setting, it conferred a resistance profile similar to VIM, IMP, and NDM [37,38]. However, beyond research contexts in recombinant clones or specific Alaskan origins, we could not find any published evidence of its widespread dissemination. Therefore, detecting *bla_LRA_* in our soil samples supports the notion of their abundance in extreme environments [38].

The higher presence of *mexF*, *mexB*, and *mexW* has been linked to chemically polluted sites (petroleum and heavy metals) [39]. In some clinically relevant *P. aeruginosa* strains, *mexB* and *mexF* may play a crucial role in their hyper-virulence and pathogenicity [40]. It has been suggested that the enrichment of bacteria capable of metabolising petroleum may contribute to the increase in the abundance of these genes. The genus *Alcanivorax*, an indigenous hydrocarbon-degrading bacterium, was identified in K1 and to a greater extent in K2 coastal soil, exhibiting the highest level of resistance among the microbial isolates. Kuwait ranks among the major oil-producing nations globally, with the petroleum sector serving as the primary industry (https://www.kockw.com/). Thus, the presence of *Alcanivorax* species appears to correlate with the occurrence of polycyclic aromatic hydrocarbons (PAHs) [41] in the region’s alkaline and saline sediments [42]. The capacity of *Alcanivorax* species to degrade persistent organic pollutants has been linked to horizontal gene transfer and a high degree of genomic plasticity [43]. This adaptability may explain the detection of *Alcanivorax* in K2 soil, which exhibited the highest observed resistance levels, suggesting potential ecological or functional specialisation [44] within this site’s microbial community. The sustained prevalence of *mexF* and *mexK* as dominant ARGs in the K2 sample points to an ongoing selection for multidrug-resistant phenotypes. However, the detection of *ugd*, specifically in soil, is a novel finding supporting the broader notion that soils can harbour resistance to polymyxins. We found no published study in soil-resistome surveys that explicitly lists the *ugd* in soil microbiota. This suggests that the presence of *ugd* in the K2 sample may represent a less-explored or under-documented phenomenon of soil resistomes. *ugd* presence is possibly linked to anthropogenic polymyxin exposure (animal manure and human activities) or co-selection pathways [45]. Other environmental resistome studies [46] highlighted *ugd* horizontal gene transfer and movement in an environmental–human interface context in a wet market environment, while Zhao et al. [47] observed a partial overlap between soil and human gut antimicrobial peptide resistomes and proposed potential gene transfer pathways; however, they did not address the *ugd* gene. In our study, we cannot provide detailed information on whether the gene is on a mobile genetic element; nevertheless, the detection of the *ugd* gene in soil is concerning, as it indicates the potential for horizontal gene transfer to human pathogens through the food chain, which could undermine the effectiveness of polymyxin antibiotics [48]. The K2 soil sample was collected from Al-Khiran, a coastal area located near Al-Wafra in southern Kuwait (Figure 1). While not primarily agricultural, its geographical position and marine influence may have indirect implications for soil characteristics and environmental conditions.

The K3 soil from Northern Kuwait, an area with intensive agricultural activity, presented a different resistance profile. While multidrug resistance genes (*mexF* and *mexW*) were still prevalent, the most abundant ARGs were *novA* and *ileS*, associated with resistance to novobiocin and mupirocin in *Geodermatophilus* [49], found in arid conditions. Within this cohort of ARGs, the plasmid-mediated quinolone resistance gene, *qepA*, is most clinically relevant. Also of clinical relevance is the detection of the *bla*_FOX-7_ and *bla*_MOX-7_ genes, expressing subgroups of AmpC-type β-lactamases, conferring resistance to a broad range of β-lactams. *bla*_FOX-7_ was found in *Aeromonas allosaccharophila*, a fish pathogen on conjugative plasmids [50]. It was later identified in Enterobacteriaceae, causing outbreaks in hospitals [51]. *bla*_MOX-7_ has been predominantly identified in *Aeromonas* sp. and in wastewater [52,53]. The distribution of *bla_Fox_* and *bla_Mox_* in soil, like other AmpC β-lactamases, may be linked to animal manure as fertilisers, antibiotics [54] and wastewater [55] usage in farming. Another notable finding in this cohort is the chromosomally located *bla_OXA_*_-18_, which was first observed in *Pseudomonas aeruginosa* and encodes a class D clavulanic acid-inhibited extended-spectrum β-lactamase [56]. Environmental surveillance data for this gene remains limited. *bla_OXA_*_-18_ is not known to be widely transmissible and has so far been reported only in clinical isolates, with no previously published evidence documenting its presence in wastewater, soil, or other environmental reservoirs. These unique findings suggest a broader range of resistance mechanisms in K3, indicating different environmental exposures or microbial interactions, and adaptation to specific antimicrobial pressures, possibly contributed by organic fertilisers from animal manure [57]. One of the reasons that rare genes may not be detected using metagenomics analysis may be that environmental AMR monitoring often targets high-priority or widely distributed ARGs [58,59].

The differentiation in both microbial community structure and ARG profiles across the three samples highlights the influence of local environmental factors (managed vs. unmanaged) on microbial population and resistance genes (Liu et al., 2021 [60]). These conditions support a closer similarity between the K1 and K3 soil samples in terms of agricultural and livestock farming activities. In contrast, the reduced diversity in the K2 sample may be due to site-specific conditions such as unmanaged soil composition, moisture content, or anthropogenic pressures that limit microbial diversity. The present study was designed as a descriptive and exploratory comparative analysis of microbiome and resistome profiles across selected sites. However, we acknowledge that without accompanying soil physicochemical data, we cannot directly attribute observed differences to specific environmental determinants. Future studies integrating detailed soil characteristics and a larger sampling size are necessary to identify the mechanisms underlying the observed differences, strengthen the study’s statistical power and improve the reliability and generalizability.

In conclusion, consistent with other soil microbiome reports, we detected an array of tetracycline resistance genes (e.g., *tet*A), aminoglycoside resistance genes (e.g., *aac(3)-Ib*) and sulphonamide resistance genes (*sul*1). The predominance of efflux pump-associated ARGs across all samples likely reflects the widespread nature of multidrug resistance in these soils, potentially sustained by natural antimicrobial compounds or anthropogenic contamination. In contrast, the presence of distinct, clinically relevant resistance genes (e.g., *ugd*, *bla_oxa_*_-18_, *bla_CMY_*_-19_, *bla_Mox_*_-7_, *bla_Fox_*_-7_, *bla_LRA_*_-12_, *novA*) suggests the influence of localised and site-specific or extreme selection pressures. This study is constrained by its focus on ARG sequences alone; consequently, important aspects such as ARG variants, mobility, and associations with human pathogens remain insufficiently resolved. Nevertheless, characterising soil microbial community structure and resistome composition is crucial for understanding the environmental reservoir of resistance and for mitigating the risk of ARG dissemination into clinical settings. Although the sample size in this study is too limited to permit broad generalisations, the identification of certain ARGs appears comparatively rare and unreported, suggesting either novel selection or unique local conditions.

## Figures and Tables

**Table 1:** Key differences between K1, K2, K3 soil samples.

Feature	K1	K2	K3
Highest Resistome Mechanism	Multidrug (efflux pump)	Multidrug (efflux pump), some polymyxin	Multidrug (efflux pump), novobiocin, mupirocin
Most Abundant ARGs	*mexF* (23%), *muxB*, *mexW*, *bacA*, *rosB*, *macB*, *mexB*, *gepA*, *bla _CM_*_19_, *bla_LRA_*, *tetA*, *aac(3)IIIb*	*mexF*, *mexK*, *ugd* (polymyxin), *qep*A, *bla*_CTXM-30_, *bla* _CTXM-123_, *tet*A.	*mexF*, *mexW*, *novA*, *ileS*, *qepA*, *bla_Fox-_*_7_, *bla_Oxa-_*_18_, *blamox_-_*_7_, *bla_LRA-_*_12_, *tetA*, *aac(3)IIIb*
ARG Profile Highlights	Efflux pump genes dominate	Multidrug + polymyxin resistance	Novobiocin and mupirocin resistance elevated
Dominant Microbial Taxa	Proteobacteria, *Alcanivorax*	*Alcanivorax*	*Geodermatophilus*
Unique Features	Highest *mexF* abundance	*ugd* gene (polymyxin)	*novA* and *ileS* highly abundant, novobiocin resistance is prominent

## Data Availability

The original contributions presented in this study are included in the article. Further inquiries can be directed to the corresponding author.
